# Cotton leaf segmentation with composite backbone architecture combining convolution and attention

**DOI:** 10.3389/fpls.2023.1111175

**Published:** 2023-01-31

**Authors:** Jingkun Yan, Tianying Yan, Weixin Ye, Xin Lv, Pan Gao, Wei Xu

**Affiliations:** ^1^ College of Information Science and Technology, Shihezi University, Shihezi, China; ^2^ National-Local Joint Engineering Research Center for Agricultural Big Data, Xinjiang Production and Construction Group, Shihezi, China; ^3^ College of Agriculture, Shihezi University, Shihezi, China

**Keywords:** cotton leaf segmentation, composite backbone, convolutional neural network, attention mechanism, transformer

## Abstract

Plant leaf segmentation, especially leaf edge accurate recognition, is the data support for automatically measuring plant phenotypic parameters. However, adjusting the backbone in the current cutting-edge segmentation model for cotton leaf segmentation applications requires various trial and error costs (e.g., expert experience and computing costs). Thus, a simple and effective semantic segmentation architecture (our model) based on the composite backbone was proposed, considering the computational requirements of the mainstream Transformer backbone integrating attention mechanism. The composite backbone was composed of CoAtNet and Xception. CoAtNet integrated the attention mechanism of the Transformers into the convolution operation. The experimental results showed that our model outperformed the benchmark segmentation models PSPNet, DANet, CPNet, and DeepLab v3+ on the cotton leaf dataset, especially on the leaf edge segmentation (MIoU: 0.940, BIoU: 0.608). The composite backbone of our model integrated the convolution of the convolutional neural networks and the attention of the Transformers, which alleviated the computing power requirements of the Transformers under excellent performance. Our model reduces the trial and error cost of adjusting the segmentation model architecture for specific agricultural applications and provides a potential scheme for high-throughput phenotypic feature detection of plants.

## 1 Introduction

Cotton, the second largest crop after grain, is the primary raw material for daily necessities and the textile industry (Feng et al., 2022). However, biotic stress and abiotic stress existing in cotton production affect the yield and quality (Zhang et al., 2022). To ensure sustainable cotton production, breeders must identify quality varieties through continuous monitoring of cotton phenotypic traits (Ye, 2014). Budding, flowering, and boll periods are significant growth stages of cotton, which are directly reflected in cotton leaves due to the influence of nutrition, diseases, and insect pests, and thus determine the subsequent growth and yield of cotton (Mubarik et al., 2020). Breeders screen the appropriate cotton varieties during the budding, flowering, and boll period, based on estimates of plant disease resistance and yield reflected by closely related leaf phenotypic traits (e.g., Leaf Length, Leaf Area Index) (Saeed et al., 2021). Manual sampling in complex field environments is a natural way to measure cotton leaf phenotypic parameters. However, manual sampling is a labor-intensive, time-consuming, and disruptive process (Bao et al., 2021). Image segmentation of computer vision is a standard approach for non-destructive sampling samples in complex field environments. The image segmentation algorithm can automatically separate the processed samples to be processed. Therefore, image segmentation has gradually become a potential preprocessing approach of sample separation for rapidly measuring plant phenotypic parameters.

With advances in computing power (e.g., GPU), deep learning with powerful nonlinear and robust generalization ability replaces the traditional image segmentation algorithm, which highly relies on expert experience (Taghanaki et al., 2020). Generally speaking, the segmentation models based on deep learning are composed of encoders and decoders, such as PSPNet (Zhao et al., 2017), DANet (Fu et al., 2019), CPNet (Yu et al., 2020), DeepLab v3+ (Chen et al., 2018). Specifically, the backbones of the segmentation models in the encoder are used to extract features (Miao et al., 2020). The feature diversity of backbone extraction determines the performance of the segmentation model (Minaee et al., 2022). Currently, convolutional neural networks (CNNs, e.g., ResNet-101, Xception) with deep stacked convolution structures to represent powerful features have gradually become mainstream feature extractors. PSPNet utilizes ResNet-101 as a backbone to achieve an elegant expression in the complex field environment of grape segmentation (Chen et al., 2021). DeepLab v3+ employs ResNet-101/Xception as a backbone to segment fruit plaques (Li et al., 2022b; Yuan et al., 2022), and also attempts to segment cotton roots (Kang et al., 2021).

CNNs have been widely used in plant phenotype, especially phenotype segmentation. However, CNNs have apparent disadvantages, such as poor learning ability of low-level features of images and partial neglect of global information, which limit the accurate segmentation of object edges in complex field environments (Liu et al., 2018). Due to the complexity of the leaf environment, the morphological characteristics (texture, size, and shape) of the leaf change accordingly, and the segmentation of the leaf edge has the dilemma of over-segmentation/under-segmentation (Yang et al., 2020). Transformers, as attention models, achieve powerful accuracy for large-scale datasets with a robust representation of global context (Dosovitskiy et al., 2021). In contrast, CNNs with deep stacked convolution structures embedded in the attention modules, e.g., Channel Attention Module (Woo et al., 2018), and Convolution Block Attention Module (Woo et al., 2018), integrate global information to a limited extent, and improve the power slightly of object edge segmentation. Thus, with the success of self-attention models such as Transformers, much previous work has attempted to bring the power of attention to computer vision (Khan et al., 2022).

Recently, Transformer-based backbones have shown potential performance and expanded cutting-edge applications. Li et al. (2022a) proposed an automatic pest recognition method based on Vision Transformer (ViT) in PlantVillage (a public dataset of plant pests and diseases) (Hughes and Salathé, 2015). Reedha et al. (2022) proposed a novel crop recognition model using ViT based on unmanned aerial vehicles (UAV) remote sensing images. Wu et al. (2021) proposed a multi-scale feature extraction model based on a visual converter to identify crop disease types. However, the large model capacity with huge parameters and high computational power required by Transformers hinders rapid application to agricultural tasks (Khan et al., 2022). The attention of Transformers has slight inductive bias and weak generalization on the relatively small amount of datasets compared with the convolution of CNNs (Dosovitskiy et al., 2021).

In relatively small agricultural data sets, plant phenotype researchers have used the Transformer and CNN cascade model, incorporating the inductive bias of CNNs and the self-attention mechanism of Transformers, to study plant phenotype. Wang et al. (2022) proposed a crop segmentation method of remote sensing images based on a barely remote sensing dataset by constructing a novel architecture of coupling CNN and Transformer. Liu et al. (2022) attempted to propose a CNN-Transformer network with Multi-Scale Context Aggregation (MSCANet) and realize efficient and effective farmland change detection. However, Transformer and CNN cascade models integrate the respective advantages of Transformers and CNNs, and the computational cost and data requirements of Transformers are also introduced into the cascade models, which hinders the rapid promotion of the cascade models in agriculture. Therefore, for the global learning potential of the self-attention mechanism of Transformers and the fast application limitation of Transformers required computing power and large-scale datasets, the models combining convolution of CNNs and self-attention of Transformers have become a new research direction. CoAtNet (Dai et al., 2021), as a novel backbone, incorporates the global awareness of Transformers and the inductive bias of CNNs.

Different from Transformer and CNN Cascade Models, CoAtNet introduces CNN convolution and Transformer attention to alleviate computational power greed. The classification speed and accuracy of CoAtNet in ImageNet demonstrate the potential of CoAtNet as a backbone for segmentation models. However, the robust backbone design of the segmentation models requires substantial trial-and-error costs (e.g., expert experience and computational costs). As the backbone architecture of automatic search, neural architecture search (NAS) (Zoph and Le, 2017) still has the computational cost of architecture search. Therefore, for backbone design, simple and effective strategies are urgently needed for rapid application in agriculture. CBNet (Liu et al., 2020) and CBNetV2 (Liang et al., 2021) proposed the architectures integrating multiple backbones into a composite backbone for object detection, which assembles multiple existing backbones in parallel to represent various features, reducing the computational cost of architecture design. Inspired by CBNet and CBNetV2, a leaf segmentation architecture based on composite backbone architecture was proposed and explored.

To the best of our knowledge, the encoder-decoder architecture segmentation model has over-segmentation and under-segmentation in complex field environments. Among the encoders of the segmentation models, the design of a robust backbone can alleviate segmentation anomalies, especially the mainstream CNNs and Transformers. CNNs are highlighted by inductive learning and generalization, while Transformers are highlighted by global semantics. However, Transformers and cotton-leaf segmentation architecture design is power consumption. Therefore, this work aims to explore the application of the composite backbone architecture combined with the convolution of CNNs and the attention of Transformers in cotton leaf segmentation without significantly introducing the computational power requirements of Transformers. The specific objectives achieved herein are as follows:

(1) Eight hundred images of budding, flowering, and boll period cotton leaves in five typical complex field environments (normal, spotted lesions, regional lesions, occluded blades, uneven illumination) were collected and labeled.
(2) CoAtNet, which incorporates the attention mechanism of Transformers into the convolution, was explored as the backbone of the encoder in the cotton leaf segmentation architecture.
(3) A simple and effective composite backbone (Xception and CoAtNet) leaf segmentation architecture combining convolution and attention was designed to fully learn the edge information and global context of cotton leaves.

**Figure 1:**
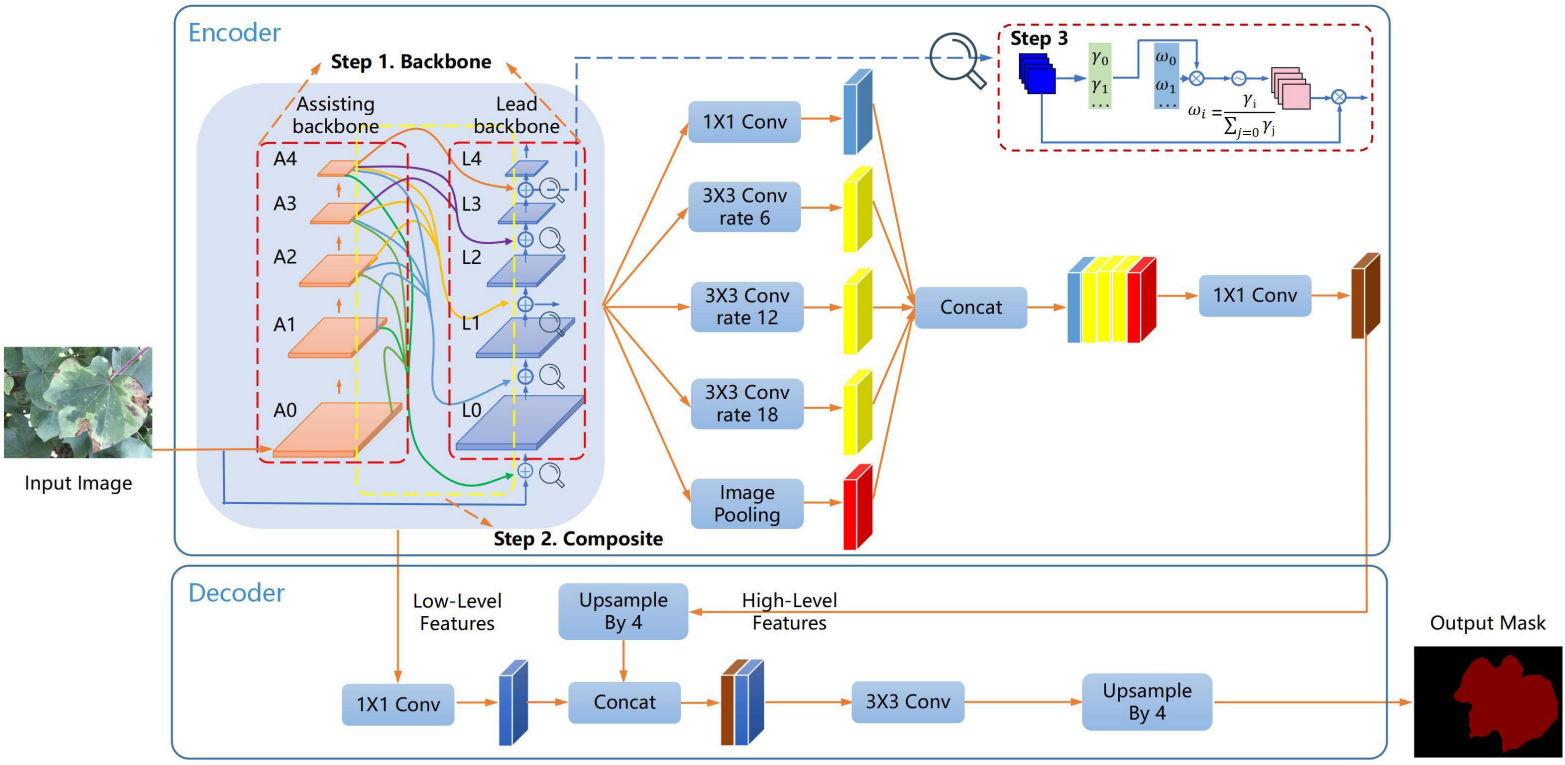
The outline of the composite backbone and segmentation architecture in our model. In step 1, the composite backbone (Xception and CoAtNet) is selected, and the features of the input image are extracted by CoAtNet. In step 2, multi-scale features are interacted in the composite backbone. In step 3, features from the composite backbone are fused using weight contribution factors.

An outline is employed to show the detailed steps of this work in **Figure 1**. Our model is based on the encoder-decoder architecture of DeepLab v3+, and the composite backbone is introduced into our model. In step 1, Xception and CoAtNet are used as the lead backbone and assisting backbone in the composite backbone, and the features of the input image are first extracted by assisting backbone. In step 2, the output features of each stage of the assisting backbone flow to parallel and lower stages of the lead backbone. Xception learns the richer multi-level features of the assisting backbone. In step 3, the fusion mechanism of weight contribution factors is adopted to suppress unimportant features from different backbones. The fused features flow to the lead backbone under the batch-normalized channel weight contribution factor. Finally, the output of the composite backbone is applied to the encoder and decoder.

## 2 Materials and methods

In this section, Section 2.1 introduced the subdivision of the cotton dataset into acquisition and preprocessing. Then, Section 2.2 illustrated the design of the segmentation model, including the model framework and the composite backbone. Finally, Section 2.3 introduced the experimental details, including the experimental structure, training, and testing strategy.

### 2.1 Data description

#### 2.1.1 Acquisition

**Figure 2:**
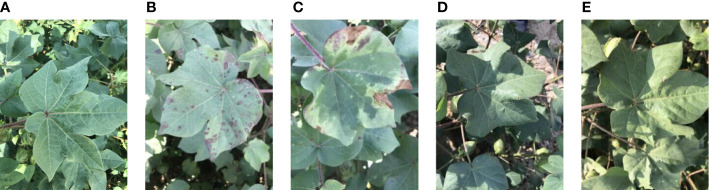
Images of the Cotton Leaf dataset. The dataset is divided into five representative leaves: **(A)** a normal cotton leaf, **(B)** a cotton leaf with spotted lesions, **(C)** a cotton leaf with regional lesions, **(D)** a cotton leaf with occluded blades, and **(E)** a cotton leaf with uneven illumination.

Cotton crops were grown in the field at the experimental station (85°9′51.231 00′′E, 44°35′47.720 00′′N) of the Agricultural College of Shihezi University, Shihezi, China. The cotton variety “Xinluzao 54” was trial-planted on April 7, 2021, and the sowing density was ten seeds/square meter. Specifically, the column spacing was 0.2 m, and the row spacing was 0.3 m. The images were acquired along the rows over the entire field on six experimental dates in the budding, flowering and boll period (June 11, June 18, June 23, July 7, July 13, and July 22). Multiple smartphones were selected to capture images and verify the generality of the subsequent segmentation models. The smartphone cameras were set to manual operation mode, with a distance of about 0.3 m from the target leaves. Specifically, the target leaves were photographed in natural light (9:00-12:00 a.m., Beijing Time). The following five types of cotton leaves were typical research objects, as shown in **Figure 2**.

- Normal leaves;
- Leaves with spotted lesions;
- Leaves with regional lesions;
- Leaves with occluded blades;
- Leaves with uneven illumination.

#### 2.1.2 Preprocess

**Figure 3:**
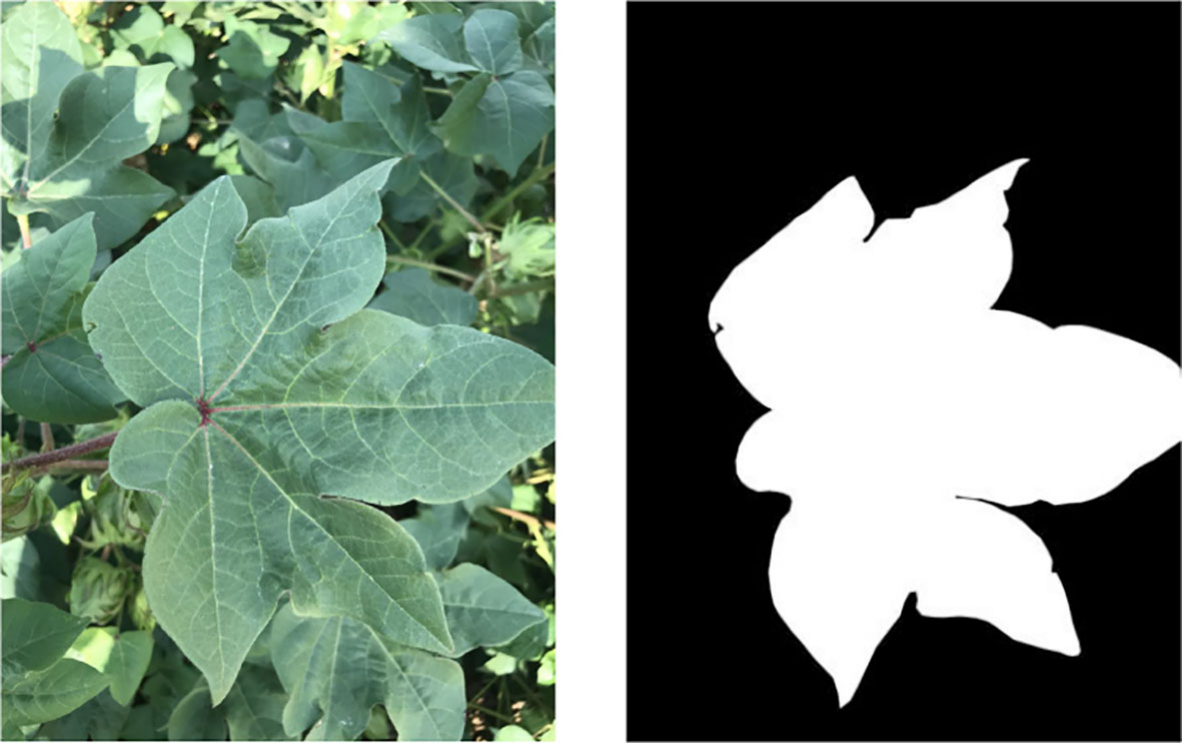
Image annotation process. The left is the input image, and the right is the labeled image.

The median filtering algorithm was applied to image preprocessing since a certain amount of image noise caused by external factors would negatively impact the training of segmentation models. Moreover, the image resolution was adjusted to 512×512 pixels before annotation, saving computational resources and labor handling time. Subsequently, the polygons pattern in Labelme-3.3.6 (Torralba et al., 2010) provided labels for two semantic classes of the dataset, including foreground (target leaves) and background (i.e., soil, weeds, other leaves). The image annotation process is shown in **Figure 3**. The diversity of leaf images under different growth periods was considered, and at least 100 images were labeled from five typical cotton leaves in the budding, flowering, and boll period.

The size and diversity of the dataset affect the segmentation model performance (Barbedo, 2018). Specifically, large-scale datasets are a prerequisite for building reliable segmentation models, while limited datasets easily lead to model overfitting. Therefore, a series of operations was adopted to expand the cotton leaf dataset: rotation and mirror flip. The final cotton leaf dataset containing 800 images and segmentation labels was divided into 80% training dataset and 20% testing dataset for training and testing subsequent segmentation models.

### 2.2 Model design

#### 2.2.1 Framework

Currently, the performing segmentation models rely heavily on the backbones. Intuitively, the rich feature maps extracted by the backbones and the vast receptive fields sensed by the backbones determine the segmentation model performance (Ma et al., 2020). However, designing and pre-training a new backbone consumes various computing resources, and requires a large number of training samples (Bao et al., 2022). Recently, the application of composite backbone in object detection has inspired our model (Liang et al., 2021). A composite backbone combines several existing networks and then integrates the rich features of multiple scales. In addition, previous studies have shown that the feature pyramid network (FPN) is more effective than simple network deepening or broadening. Top-down paths of FPN introduce spatially richer and semantically more powerful high-level features and enhance low-level features in bottom-up paths of FPN. Thus, in our model, multiple backbones are composited and called assisting backbone and lead backbone, respectively. The composite backbone of our model extended FPN (Lin et al., 2017) idea combines high-level and low-level features from multiple networks.

As a classical semantic segmentation model, DeepLab v3+ (Chen et al., 2018) is used as the benchmark for segmentation models. Therefore, DeepLab v3+ is regarded as the prototype of our model, and the lead backbone is the Xception applicable to segmentation in the raw DeepLab v3+. However, DeepLab v3+ still does not fully show excellent potential performance and only tries mature convolutional neural networks (CNN) as a backbone. As a Backbone, simple CNN has the problems of missing global information and tiny local receptive fields, which cannot meet the requirements of DeepLab v3+ for feature maps. In addition, CoAtNet (Dai et al., 2021) integrates the attention mechanism of Transformers into the convolution operation of CNN, maintaining the optimal tradeoff between model generalization capability and model capacity. Therefore, the hybrid family of CoAtNet is used as the assisting backbone of our model (based on DeepLab v3+).

As shown in **Figure 1**, our model is based on the encoder-decoder architecture of DeepLab v3+. Our model uses Xception (Chen et al., 2018) and CoAtNet (Dai et al., 2021) as the lead backbone and assisting backbone. In addition, our model is inspired by FPN and contains long-skip connections from the encoding path to the decoding path and short-skip connections between the composite backbone. Long-skip connections transmit low-level features and high-level features. Short-skip connections fuse assisting backbone and lead backbone features, and transmit to the lead backbone.

The remaining parts retain the original architecture of DeepLab v3+. The encoder of the atrous spatial pyramid pool (Chen et al., 2017) module processes the lead backbone output features with five different operations, namely 1×1 convolution, 3×3 convolution at dilation rate 6, 3×3 convolution at dilation rate 12, 3×3 convolution at dilation rate 18, and Image Pooling. The output features of five different operations are downsampled to 1/16 of the input image size and then combined to form multi-scale features. The multi-scale features are then subjected to 1×1 convolution operation to form high-level features. The low-level features output by the assisting backbone A1 are combined and fused with the high-level features four times up-sampled after the 1×1 convolution operation. The low and high-level fusion features are restored to the input image size by 3×3 convolution and four times upsampling. In our model, two dropout layers are added before the last four times upsampling layers to avoid overfitting. The softmax function finally activates our model. Each channel value of the activation output represents the category probability, and the maximum probability value determines the pixel category.

#### 2.2.2 Backbone

**Figure 4:**
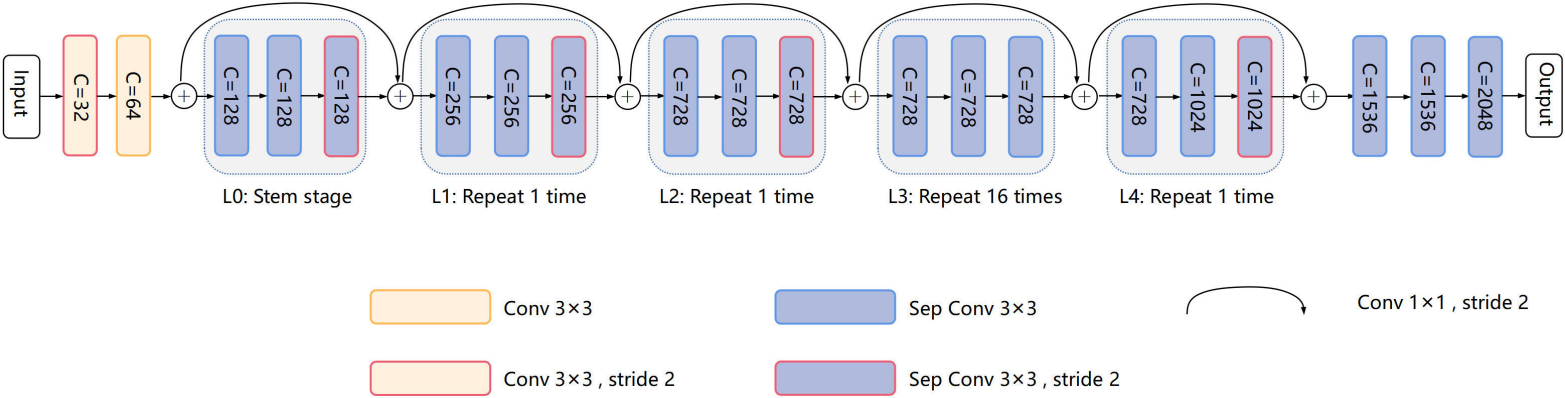
Xception. C represents the number of feature output channels.

Our model is based on CoAtNet and Xception as the composite backbone. As shown in **Figure 4**, the official Xception backbone for segmentation is retained as the lead backbone. In our model, the lead backbone and assisting backbone are divided into five standard blocks, which are L0, L1, L2, L3, and L4 of the lead backbone, and A0, A1, A2, A3, and A4 of the assisting backbone in turn. Concretely, our model divides Xception into five modules, L0, L1, L2, L3, and L4, according to the remaining residual connection after the first residual connection. Modules L0, L1, L2, L3, and L4 are composed of only 3×3 separable convolution to reduce computational power requirements. The L3 module is repeated 16 times to learn the image features fully. The rest consists of 3×3 convolution and 3×3 separable convolution. 1×1 convolution achieves feature channel rise and residual transfer. In Xception, the number of channels of the feature map increases successively, and the partial convolution step is set to 2 to fully capture the spatial information of the feature map and reduce the spatial resolution.

**Figure 5:**
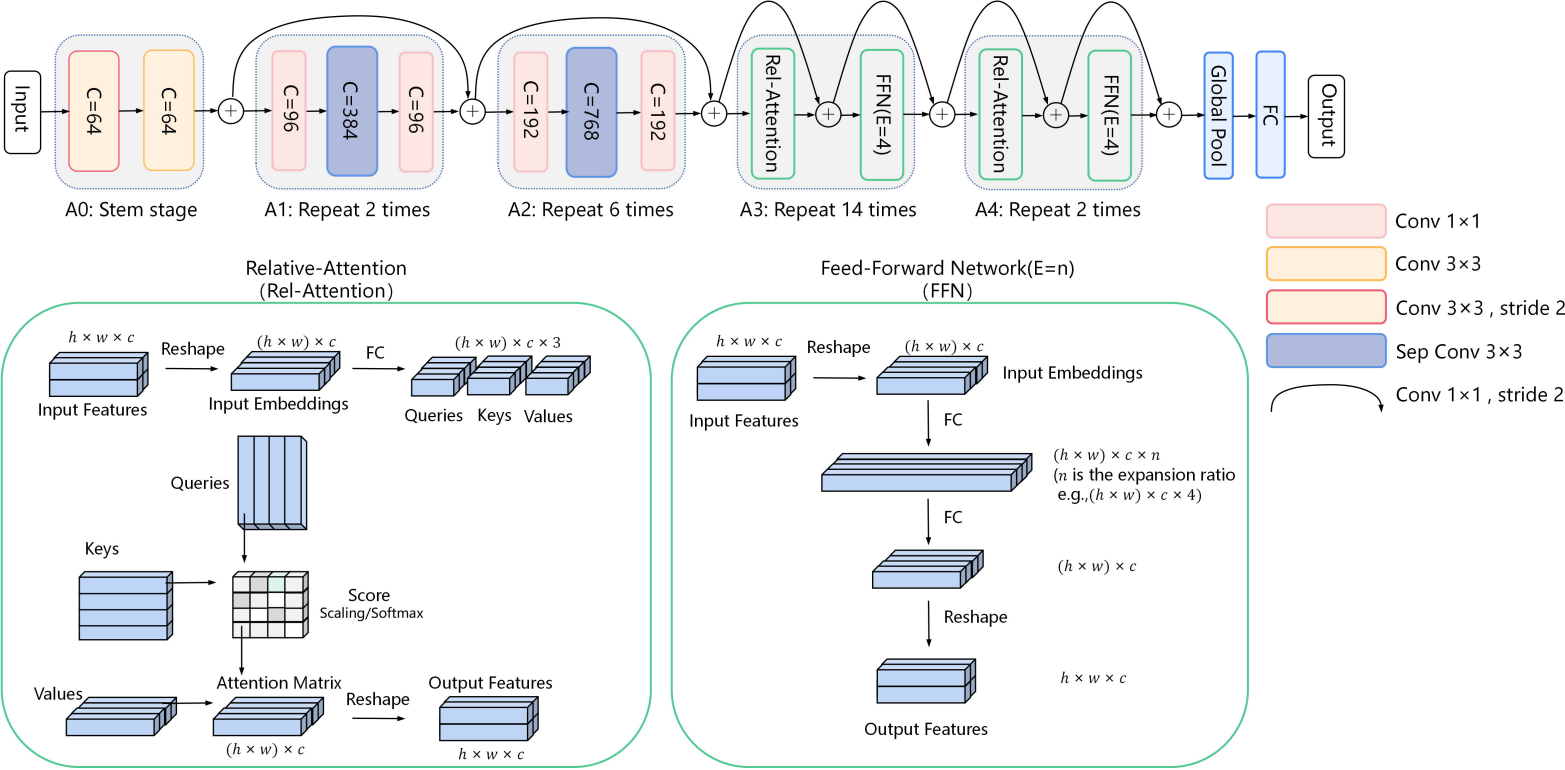
CoAtNet. CoAtNet is divided into five modules: three convolution modules, A0, A1, A2, and two self-attention modules, A3 and A4. C represents the number of feature output channels. E is the n-time expansion rate of the Feed-Forward Network (FFN) layer.

As shown in **Figure 5**, in our model, the assisting backbone consists of three convolution modules, A0, A1, and A2, and two self-attention modules, A3 and A4. The A0 module consists only of 3×3 convolution, which reduces the feature spatial resolution. Modules A1 and A2 are expanded by the attention mechanism of MobileNet consisting of 1×1 convolution and 3×3 separable convolution (MBConv module with inverted bottleneck structure) (Sandler et al., 2018). 1×1 convolution is used to increase and reduce the dimension of the feature. A3 and A4 modules contain a Relative-Attention (Rel-Attention) layer and a Feed-Forward Network (FFN) layer for learning global feature information. The modules A1, A2, A3, and A4, are successively repeated 2, 4, 8, and 2 times to explore the features fully. The rest consists of global pooling and a fully connected (FC) layer. The residual connection is guaranteed to reduce the model complexity to reduce overfitting, while the residual connection prevents the gradient from disappearing. Specifically, 1×1 convolution carries out feature channel dimension raising and completes the residual transfer.

The Rel-Attention layer expands the attention mechanism of Transformers. The Rel-Attention layer stretches the input features from three-dimensional to two-dimensional, that is, h×w×c to (h×w)×c, and then gets the Input Embeddings. The trainable weight matrices of Queries, Keys, and Values are calculated by the Input Embeddings with the full connection. Intuitively, the two-dimensional matrix Queries, Keys, and Values all contain feature global information. The Score matrix is computed by the scalar product of Queries and Keys. The Score matrix represents the correlation between each one-dimensional vector in Keys and each one-dimensional vector in Queries. Further, the Score matrix is scaled and activated by the softmax function. Then, the Attention Matrix is obtained by calculating the scalar product between the Score matrix and Values, which contain relative global attention features of each one-dimensional vector in the three matrices of Queries, Keys, and Values. Finally, the Attention Matrix is reconverted into three dimensions to obtain the output features.

The FFN layer learns advanced image features from the MBConv block. The Input Embeddings are expanded by an FFN layer consisting of multiple FC layers with an n- time expansion rate and then resized to the original size. In our model, the number of feature channels in the FFN layer inflation factor was set to 4.

#### 2.2.3 Composite

**Figure 6:**
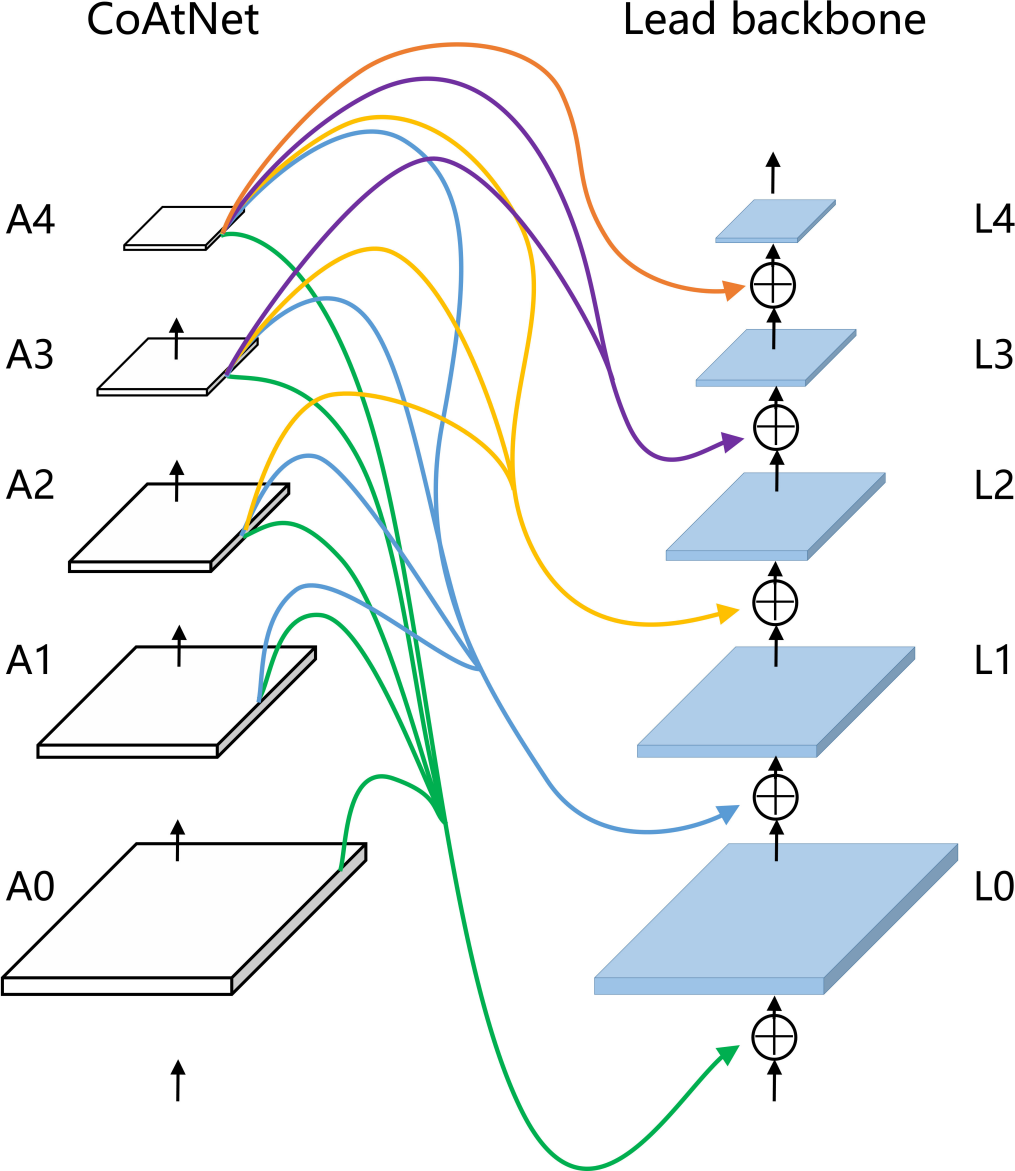
Our composite backbone architecture with CoAtNet as assisting backbone.

Backbone, or feature extractor, as the initial stage of the semantic segmentation network, plays a significant role in model segmentation performance (Fan et al., 2018). Backbone provides the basic features of the segmentation target for the semantic segmentation model. Our model draws on the ideas of FPN (Lin et al., 2017) and CBNetV2 (Liang et al., 2021) architecture to construct the connection structure between the lead backbone and the assisting backbone. As shown in **Figure 6**, the output features of modules A0, A1, A2, A3, and A4 of CoAtNet flow to parallel and lower-level jump connections of Xception. Xception both preserves the original residual connection and learns the richer multi-level features of the assisting backbone. Specifically, the output feature maps of modules A0, A1, A2, A3, and A4 are consistent with the dimension of the output feature maps of Xception and skip-connections of lower stages by 1×1 convolution. Subsequently, linear interpolation keeps the output feature maps of A0, A1, A2, A3, and A4 modules consistent with the spatial resolution of the output feature maps at parallel and lower skip-connections of Xception. Finally, the output feature maps of modules A0, A1, A2, A3, and A4 are element-summed with the output feature maps at parallel and lower-level skip-connections of Xception.

**Figure 7:**
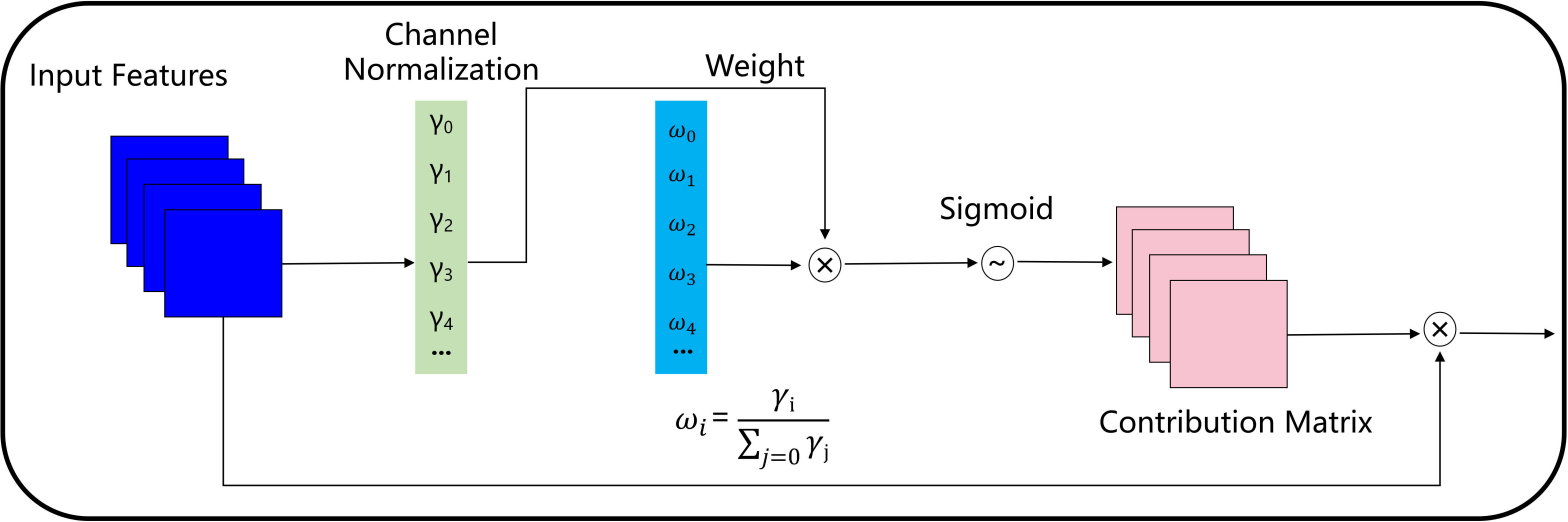
Fusion mechanism of weight contribution factors based on batch normalization. Where, *γ*_*i*_ represents the weight value of the i-th channel calculated in batch normalization, *γ*_*j*_ represents the weight value of the j-th channel calculated in batch normalization, *ω*_*i*_ represents the importance degree of the i-th channel.

The output of each stage of the assisting backbone flows to parallel and lower stages of the lead backbone. The output of the lead backbone is applied to downstream tasks. Different from the simple network deepening or broadening, the composite backbone, which integrates the high and low-level features of the composite backbone, gradually expands the receiving field and provides richer target information. Due to the different response values of the multi-level features integrating the composite backbone, the model is prone to convergence dilemmas. Inspired by the accelerated convergence of normalization (Yan et al., 2020), our model adopts the fusion mechanism of weight contribution factors to suppress unimportant features, as shown in **Figure 7**. The fused features flow to the lead backbone of Xception under the batch-normalized channel weight contribution factor.

### 2.3 Experiment

#### 2.3.1 Experimental detail

##### 2.3.1.1 Hardware

Experiments were conducted with the following hardware configurations: Intel(R) Core(TM) i7-11700 K CPU, 128GB memory, and NVIDIA GeForce RTX3090 graphics card.

##### 2.3.1.2 Software

The deep learning framework PyTorch installed in Windows 10 (Microsoft, United States) was adopted to build neural network models.

##### 2.3.1.3 Loss function

Models were optimized by the cross-entropy loss (cost) function (Huang et al., 2016). As shown in Equation (1), *y*_*i*_ represents the label of the pixel, *p*_*i*_ represents the predicted value of the pixel, and *m* represents the number of pixels in the image.


(1) $$Loss=\sum_{i=1}^{m}-\left(y_{i}\log\left(p_{i}\right)+\left(1-y_{i}\right)\log\left(1-p_{i}\right)\right)$$

The composite backbone was applied in our model to train the original cross-entropy loss. The assisting backbone, which inherited the assistant loss concept of CPNet, was also used to produce assistant supervision. In other words, original cross-entropy loss bears the greatest responsibility, and assistant supervision helps to optimize the learning process. Meanwhile, super parameter weight was added to balance the assistant supervision. The loss defined in our model is as Equation (2).


(2) $$L=L_{Comp}+\lambda \cdot L_{Assist}$$

Where *L*_*Comp*_ is the loss of the composite backbone from input to output,is the loss of assisting backbone from the input only through the low-feature path to the output, and *λ* is the super parameter weight for the assistant supervision. In our model, *λ* was set to 0.3 according to our empirical experiments.

#### 2.3.2 Training strategy

Two training strategies were used on the cotton leaf dataset for our model. In the first strategy, our model was trained from scratch. In the second strategy, to use the leaf information of the source domain and effectively transfer knowledge to the target domain, the PlantVillage (Hughes and Salathé, 2015) dataset consisting of crop leaf images was first used to pre-train the lead backbone and the assisting backbone. The composite backbone with pre-trained weights in a fine-tuning paradigm of the training process to achieve fast learning on the cotton leaf dataset. In particular, in the fine-tuning paradigm, the composite backbones were frozen to train the encoder-decoder part of our model fully. Then, the composite backbones were unfrozen to complete the rest after the model was trained for a certain epoch.

The parameter setting in training from scratch is shown in **Table 1**, and the parameter setting in fine-tuning is shown in **Table 2**. The optimizer of our model was the adaptive moment estimation optimizer (Adam) (Kingma and Ba, 2015). In Adam, the first and second moments of the gradient were used to update and correct the current learning rate (Dong et al., 2017). More importantly, if the loss did not improve for more than five epochs during the training, the minimum learning rate was set to 0. Otherwise, the learning rate would drop by 1/2, and the model would continue to train at that learning rate. The model would stop training until the loss no longer changes significantly or until the maximum number of iterations was reached.

**Table 1:** The parameter setting in training from scratch.

Optimizer	Learning rate	Batch size	Epochs
Adam	5e-4	4	200

**Table 2:** The parameter setting in fine-tuning training.

Training Stage	Optimizer	Learning rate	Batch size	Epochs
Backbone freezing	Adam	1e-4	8	100
Fine-tuning	Adam	5e-5	4	100

#### 2.3.3 Testing strategy

Pixel Accuracy (PA), Mean Pixel Accuracy (MPA), and Mean Intersection over Union (MIoU) (Shelhamer et al., 2015) are used to evaluate the effect of our model, as shown in Equation (3), (4) and (5).


(3) $$PA=\frac{\sum_{i=0}^{k}p_{ii}}{\sum_{i=0}^{k}\sum_{j=0}^{k}p_{ij}}$$

(4) $$MPA=\frac{1}{k+1}\sum_{i=0}^{k}\frac{p_{ii}}{\sum_{j=0}^{k}p_{ij}}$$

(5) $$MI\text{o}U=\frac{1}{k+1}\sum_{i=0}^{k}\frac{p_{ii}}{\sum_{j=0}^{k}p_{ij}+\sum_{j=0}^{k}p_{ji}-p_{ii}}$$

Where, *k* represents the number of classes, *i* represents the true value, *j* represents the predicted value, and *p*_*ij*_ represents the pixels that predict class *i* as class *j* . Generally, *p*_*ii*_ represents real samples (TP), *p*_*ij*_ represents false negative samples (FN), and *p*_*ji*_ represents false-positive samples (FP).

However, the MIoU score is higher than the true value when measuring the boundary quality, which cannot gracefully evaluate the segmentation results of our model. Accordingly, Boundary Intersection over Union (BIoU) is introduced as an additional evaluation metric to compare the segmentation fineness better (Cheng et al., 2021). BIoU is used to evaluate the boundary quality of segmented objects based on the sensitivity of boundary error. BIoU is defined as Equation (6).


(6) $$BI\text{o}U=\frac{\left|\left(G_{d}\cap G\right)\cap \left(P_{d}\cap P\right)\right|}{\left|\left(G_{d}\cap G\right)\cup \left(P_{d}\cap P\right)\right|}$$

Where *G* denotes the ground truth binary mask, *P* denotes the prediction binary mask, and *d* denotes the pixel width of the boundary region. Boundary regions *G*_*d*_ and *P*_*d*_ are the sets of all pixels within *d* pixels distance from the ground truth and prediction contours, respectively.

## 3 Results and discussion

### 3.1 Segmentation model comparison experiment

Segmentation models adopt the experimental setting in Section 2.3.2 for training to make the comparison fair. The performance of segmentation models in training from scratch is shown in **Table 3**, and the implementation of segmentation models in fine-tuning is shown in **Table 4**.

**Table 3:** The performance of segmentation Models in training from scratch.

Method	Backbone	Multi-scale Fusion	Attention	Assistant Supervision	BIoU	MIoU	MPA	PA
PSPNet	ResNet-101	◯	×	×	0.415	0.826	0.869	0.877
DANet	ResNet-101	×	◯	×	0.488	0.883	0.917	0.933
CPNet	ResNet-101	×	×	◯	0.497	0.896	0.927	0.941
DeepLabv3+	Xception	◯	×	×	0.522	0.911	0.951	0.967
Ours	Composite (Xception + CoAtNet)	◯	◯	◯	**0.583**	**0.924**	**0.964**	**0.972**

The bold values indicate the maximum value in their columns.

**Table 4:** The performance of segmentation Models in fine-tuning training.

Method	Backbone	Multi-scale Fusion	Attention	Assistant Supervision	BIoU	MIoU	MPA	PA
PSPNet	ResNet-101	◯	×	×	0.438	0.866	0.893	0.901
DANet	ResNet-101	×	◯	×	0.513	0.899	0.925	0.943
CPNet	ResNet-101	×	×	◯	0.533	0.911	0.937	0.953
DeepLabv3+	Xception	◯	×	×	0.565	0.923	0.957	0.972
Ours	Composite (Xception + CoAtNet)	◯	◯	◯	**0.608**	**0.940**	**0.975**	**0.979**

The bold values indicate the maximum value in their columns.

Compared with the encouragement success of training from scratch, the evaluation indexes (BIoU and MIoU) of each segmentation model in fine-tuning training were improved accordingly. In addition, among the two training strategies, PSPNet fused multi-scale features to obtain the baseline effect in the cotton leaf segmentation task under complex background. DANet inherited the attention mechanism to improve the cotton leaf segmentation task. CPNet had achieved moderate results without multi-scale feature fusion and attention mechanism, considering assistant supervision strategy. DeepLab v3+ took a mature CNN (Xception) as a backbone, which was the benchmark level in several standard segmentation models, both in MIoU, which represented the overall segmentation quality of the cotton leaf, and in BIoU, which meant the segmentation quality of the leaf edge.

Our model had significant progress compared with DeepLab v3+. Specifically, among MIoU with already high ratings, our model increased by about 1%, due to data limitations or task bottlenecks with an inconspicuous rise. However, in BIoU, our model improvement was quite noticeable, with an increase of around 5%. Without loss of generality, the BIoU was enhanced due to the composite backbone (Xception + CoAtNet). The introduction of our composite backbone not only guaranteed the generalization ability and convergence ability based on Xception, but also had the global receptive field of the self-attention layer based on CoAtNet. The global information ensured that our model worked more accurately in cotton leaf edge segmentation. Due to the structure of the composite backbone, multi-level features were obtained by the encoder and decoder of our model, thus enabling the edge pixel predictor to get a rich feature map. In addition, our model considered the progress of CPNet, which also increased the weight of our assisting loss. At the same time, the composite backbone architecture retained the conventional training mode of the backbone in essence. Decoupling the composite backbone and then pre-training the weight of the individual backbones independently was low-cost.

### 3.2 Segmentation model robust experiment

**Figure 8:**
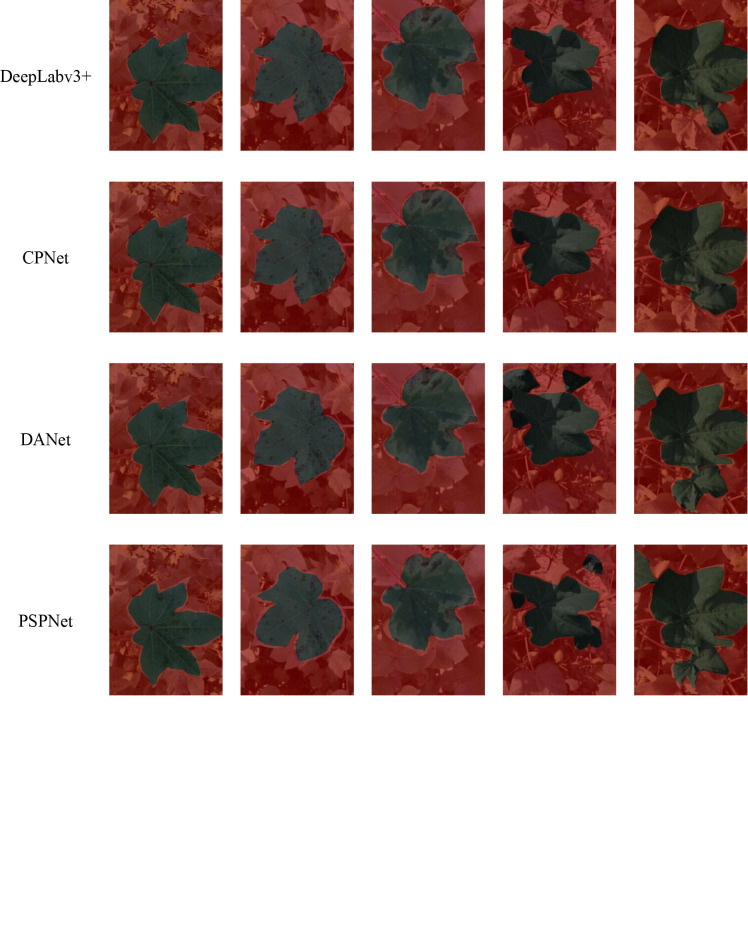
Pre-trained segmentation models results on five types of cotton leaf images.

To make the comparison concrete, various images from the test set of the cotton leaf dataset were selected to visualize the results of the pre-trained segmentation models, and the types of cotton leaf images were described in Section 2.1.1. The comparison results are shown in **Figure 8**. The segmentation models effectively detect normal and diseased cotton leaves (spotted and regional lesions), especially in detecting cotton leaf edges. The texture features and shape parameters of the cotton leaves during training were simple to learn. Under the condition of shadow occlusion, the overall segmentation of our model and DeepLab v3+ was satisfactory. At the same time, CPNet had the under-segmentation phenomenon, DANet and PSPNet had the over-segmentation and under-segmentation phenomenon. DeepLab v3+, CPNet, DANet, and PSPNet over-segmented cotton leaves compared with the segmentation acceptable to our model under uneven illumination conditions.

The PSPNet, with ResNet-101 as the backbone, incorporated multi-scale features. The segmentation of normal and diseased cotton leaves (spotted and regional lesions) was consistent with the further determination of cotton phenotypic traits. DANet integrated with the attention mechanism, similar to PSPNet, and both had under-segmentation under the condition of shadow occlusion and uneven illumination. CPNet and DeepLab v3+, in turn, due to the backbone update and the introduction of assistant losses, the overall segmentation level was moderately acceptable except for under-segmentation in shadows and over-segmentation in uneven illumination. Since the conventional segmentation models only contained the convolution module and lacked the global receptive field, the conventional segmentation models could not learn the subtle differences between pixels. The processing effects of leaf edges were poor in the complex filed environment.

In contrast, our model based on DeepLab v3+ accurately segmented cotton leaves in typical scenes, especially the edge of cotton leaves. Due to the proper coordination of convolution and self-attention module of assisting backbone CoAtNet and the penalty of assisting loss, our composite model could effectively learn the local and global context of complex background. The excellent performance of our model cannot be achieved without the self-attention module in the assisting backbone. In addition, our model inherited the idea of the various benchmark models to ensure that the encoder had full access to the information from the multi-layer features.

### 3.3 Ablation experiment

**Figure 9:**
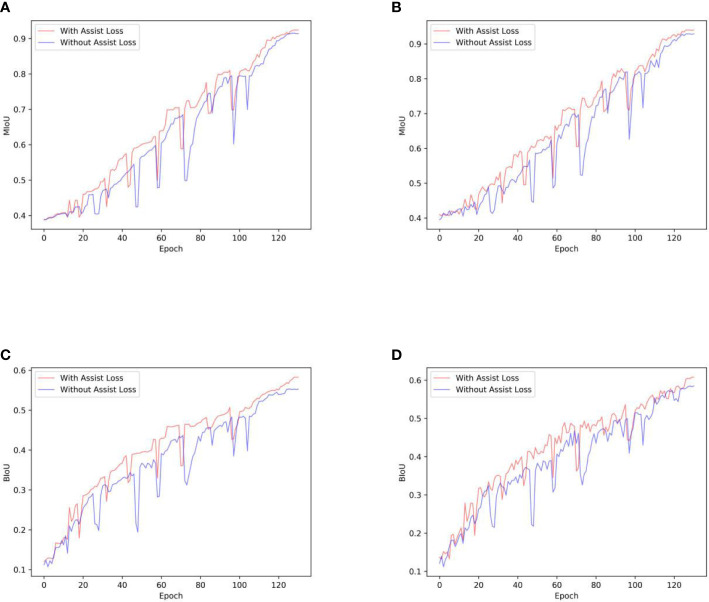
The effects of assistant supervision: **(A, C)** represent the changing trends of MIoU and BIoU in training from scratch, **(B, D)** are the changing trends of MIoU and BIoU in fine-tuning training.

The assistant supervision in our model ensured that the assisting backbone contributed to the segmentation. Therefore, the penalty of assisting loss enables the model to learn more cotton leaf features, as CPNet achieved satisfactory improvement by only considering assistant loss. In addition, to fairly compare the progress of our model with DeepLab v3+, the results of decoupled assistant supervision are shown in **Figure 9**. **Figure 9** shows the improvement effect of assistant supervision in training from scratch and fine-tuning training strategies. In the training-from-scratch strategy, MIoU and BIoU improved from 0.915 to 0.924, and 0.553 to 0.583, respectively. Accordingly, in the fine-tuning training strategy, MIoU and BIoU improved from 0.929, and 0.585 to 0.940 and 0.608, respectively.

In training from scratch and fine-tuning training strategies, the trajectory occasionally shows sudden declines. One of the reasons for the decline phenomenon may be the random loading of batch samples in the training data set to train our model. The randomness of training samples led to significant fluctuations in the parameters of our model, which further affected the performance of our model on the test dataset. Besides, to prevent the model from overfitting, two dropout layers were added before the upsampling layer of the decoder. Although the dropout layers can improve the robustness of our model, the dropout layers cause important neurons to be randomly deactivated, which would be the reason for the sudden declines of the trajectory. However, the introduction of assisted supervision promoted the segmentation power of our model, and the training was smoother than that of the non-assisted supervision strategy. The trajectory can recover and rise in fewer epochs after a sudden decline with assisted supervision. The segmentation effect of our model was suboptimal without adopting the assisted supervision strategy. Generally, the attention mechanism of Transformers integrated into the composite backbone of our model achieved remarkable results. Due to the limitation of computing resources, the computational requirements of the Transformer cannot be met. Further, in the ablation experiment, the assisting backbone is replaced by the Transformer for comparison with our model. However, our model incorporated the attention mechanism for the broad success of Transformers, which provides a feasible strategy for overcoming the computational power requirements of Transformers and applying Transformers elegantly to agricultural tasks.

## 4 Conclusion

In this work, from five typical cotton leaves (normal, spotted lesions, regional lesions, occluded blades, uneven illumination), a total of 800 images were labeled at the budding, flowering, and bolling stages. The composite backbone-based encoder and decoder semantic segmentation architecture (our model) was used for cotton leaf segmentation in complex field environments. The composite backbone consisted of the lead backbone Xception and the assisting backbone CoAtNet, saving the computational cost of architecture search for cotton-leaf segmentation. Xception represented the biased learning and generalization of CNN, CoAtNet was integrated into our model with the global context inherited from Transformers. Due to the slight computational power and data requirements of CoAtNet compared with Transformers, our model not only maintained the fast convergence of convolution but also maintained the global receptive field of attention under the constraint of a certain computational cost. At the same time, the introduction of the multi-scale feature fusion mechanism and assistant supervision strategy effectively improved the performance of our model. The experimental results showed that the cotton leaf segmentation performance of our model, especially under complex filed environments, was significantly better than that of the PSPNet, DANet, CPNet and DeepLab v3+ benchmark models, and the under-segmentation and over-segmentation of five typical cotton leaves were encouraging. In addition, different backbones can be trained offline and reassembled into composite backbones with limited computing resources. In the future, more types and numbers of pre-trained backbones can be combined to achieve faster and better plant high-throughput phenotypic tasks.

## Data availability statement

The raw data supporting the conclusions of this article will be made available by the authors, without undue reservation.

## Author contributions

JY, TY, PG, and WX contributed to conception and design of the study. JY, TY, and WY contributed to the preparation of equipment and the acquisition of data. JY and TY wrote the code and tested the method. JY, TY, WY, PG, and WX validated the results. JY wrote the first draft of the manuscript. TY, PG, WX, and XL wrote sections of the manuscript. All authors contributed to manuscript revision, read, and approved the submitted version.
